# The expression and prognostic value of transporter 1, ATP binding cassette subfamily B member in clear cell renal cell cancer with experimental validation

**DOI:** 10.3389/fonc.2022.1013790

**Published:** 2022-11-07

**Authors:** Zhen-Da Wang, Xi Tian, Yue Wang, Jun-Jie Wang, Shi-Qi Ye, Yong-Qiang Huang, Yuan-Yuan Qu, Kun Chang, Guo-Hai Shi, Ding-Wei Ye, Cheng-Yuan Gu

**Affiliations:** ^1^ Department of Urology, Fudan University Shanghai Cancer Center, Shanghai, China; ^2^ Department of Oncology, Shanghai Medical College, Fudan University, Shanghai, China

**Keywords:** clear cell renal cell carcinoma, prognosis, TAP1, tumor immune microenvironment, therapeutic biomarker

## Abstract

Transporter associated with antigen processing 1(TAP1) serves as a protein to transport antigenic peptides from the surface of the endoplasmic reticulum to the lumen of the endoplasmic reticulum when the antigens are presented by major histocompatibility complex type I (MHC-I), which has been identified to play a critical role in antigen presentation in innate immunity. In tumors, the role of TAP1 seems to remain controversial. On the one hand, given the role of TAP1 in antigen presentation, it is indicated that high TAP1 expression corresponds to the emergence of more neoantigens epitopes that facilitate the recognition for phagocytes, T cells and other cells. On the other hand, the genetic ablation of transporter associated with antigen processing (TAP) results in the presentation of new class I-restricted epitopes encoded in house-keeping products. Opposite result has been revealed by studies in other tumors suggest, which implies a more complex function of TAP1. Therefore, it’s significant to clarify the role of TAP1 in clear cell renal cell carcinoma (ccRCC). In this study, we found the elevated expression levels in mRNA and protein of TAP1 in ccRCC tissues, which indicated a relatively worse prognosis. Transwell assay and Scratch assay *in vitro* demonstrated the promotive role of TAP1 in ccRCC migration as well as a significant role in metastasis. And the increased expression of TAP1 resulted in more immune cells infiltrated in cancer tissues. TAP1 was also demonstrated to be related to immune regulator genes, as gene set enrichment analysis (GSEA) indicated its significant role in immune regulation. The results of CancerSEA indicated the positive association of the high-level TAP1 expression with epithelial–mesenchymal transition (EMT) and the inverse association with Cell Cycle. The effective drugs were also predicted based on TAP1 expression, of which the high level was indeed associated with resistance to multiple drugs, but some effective drugs still identified based on high TAP1 expression. According to the analysis of various databases, the role of TAP1 in ccRCC was explored, especially in relationship of TAP1 with tumor microenvironment. These results indicate that TAP1 can serve as a potential target for treatment of ccRCC.

## Introduction

Renal Cell Carcinoma (RCC) as one of the most common types of urinary system tumor exhibits an increasing trend of incidence. In America, about 79000 American was newly diagnosed with kidney and renal pelvis cancer in 2022 (1). There are multiple histological subtypes of renal cancer, each characterized by a unique molecular landscape, where the clear cell renal cell carcinoma(ccRCC) lies the most common type of RCC, accounting for 75% of all emerging cases, which originates from the proximal tubule cells of the kidney nephron. Up to 30% of patients receiving curative treatment for localized RCC have developed tumor recurrence after considered disease-free, and patients with metastatic ccRCC (mccRCC) display a poor prognosis, with less than 10% of patients maintained alive 5 years after metastasis (2–4). Therefore, more biological markers require to be uncovered that may involve more biological processes without appreciation previously.

Tumor microenvironment(TME) is involved in multiple components of surrounding tumor cells, especially all kinds of immune cells. CcRCC is one of the most immune-infiltrated tumors in pan-cancer comparisons (5). Immune-infiltration feature is associated to tumor biology activity and the response to immune checkpoint inhibitors (ICI) treatment. Despite a novel era of tumor therapy inaugurated by PD-1 as one of the immune checkpoint inhibitors, the efficacy of PD-1 still remains limited due to the complexity of the tumor immune escape mechanism (6). CcRCC has developed different strategies to escape T cell-mediated immune surveillance, like poor antigen-presenting ability, modulation of immune stimulatory or immune suppressive molecules and alterations in the cellular composition of the TME (7–10). Moreover, the immune microenvironment is dynamic and complex. The identification of new tumor immune-related genes is crucial for understanding the regulatory mechanism of the tumor immune microenvironment and establishing a tumor prognosis model based on immunity.

Intracellular processing of antigens is weighted for T cell immunity towards cancers, since cleaved peptides act as the molecular targets of lymphocytes (11). Transporter associated with antigen processing 1(TAP1) is considered the transporter associated with antigen-processing of major histocompatibility complex I (MHC I) for recognized by immune cells. Various studies have demonstrated that mutations or changes in the expression of TAP1 play roles in tumor immune escape and affect tumor progression, while still remain certain controversial (12–16) in terms of cancer. A considerable amount of research has focused on the relationship between polymorphisms in TAP1 and tumorigenesis development (17–19).On the one hand, in some cancers, such as colorectal cancers, researchers work to promote antigen presentation and thereby enhance tumor sensitivity to drugs (20–22). The high expression of TAP1 may be associated with the production of more tumor neoantigens, which in turn initiate tumor immunity (23). On the other hand, recently, researchers have illustrated that the silent TAP1 induced the presentation of a TAP-independent peptide in human tumor cells, and thereby enhanced the effectiveness of immune potentiating therapies (24, 25), which is additionally considered associated with tumor drug resistance (26–28). Therefore, it is necessary to uncover the role of TAP1 in ccRCC.

Here, our results indicated that the high expression of TAP1 in ccRCC promotes the tumor metastasis. It further suggested that the high expression of TAP1 in ccRCC could serve as an immune-related molecule to predict the prognosis of this disease. TAP1 may become a new promising clinical target.

## Materials and methods

### Databases to analyze TAP1 expression in human cancers

Gene Expression Profiling Interactive Analysis (GEPIA) database (http://gepia2.cancer-pku.cn/#analysis) was quoted for the comparison in TAP1 expression between Kidney renal clear cell carcinoma (KIRC) and paired normal tissue (29). The University of ALabama at Birmingham CANcer data analysis Portal (UALCAN) database (http://ualcan.path.uab.edu/index.html) was adopted for the analysis of the expression profiles of TAP1 in different cancer and paired normal cell lines (30, 31). In the Oncomine database, the thresholds was set to 0.001 for P-value and 1.5 for the fold change.

### The Human Protein Atlas

The Human Protein Atlas (HPA) (https://www.proteinatlas.org/) is an open access protein database (32). composed of proteomic data based on 26941 antibodies targeting 17165 unique proteins. The KIRC proteome data were divided into two groups according to the expression of TAP1 protein: the high expression group and the low expression group, with the survival analysis carried out.

### Immune infiltration analysis

The tumor immune estimation resource (TIMER) database (33) (http://timer.cistrome.org/) could be provided to calculate the abundance of tumor-infiltrating immune cells (TIICs) in tumor tissues with the sequencing data applied. Depending on this database, the correlation between the TAP1 gene expression and the abundance of TIICs (neutrophils, CD4+ T cells, B cells, CD8+ T cells, macrophages, and dendritic cells) were determined in 38 tumors. QuanTIseq R script was applied to quantifying the relative proportions of infiltrating immune cells, covering B cells, Macrophages M1, Macrophage M2, Monocytes, Neutrophils, NK cells, CD4+ T cells, CD8+T cells, Tregs, Dendritic cells (34). Spearman’s rank correlation analysis was performed in the exploration of the relationship between the risk score values and the immune infiltrated cells.

### Gene set enrichment analysis

To determine the downstream signaling pathways and potential biological functions of TAP1 in KIRC, the KIRC tissues were divided into high-expression and low-expression group based on the level of expression of TAP1. By employing the enrich-plot package based on the c5.all.v7.1. symbols file, gene set enrichment analysis (GSEA) was performed on the sequencing data of the KIRC samples from the The Cancer Genome Atlas (TCGA) database. Then, the enrichment maps were plotted. The results were considered statistically significant when p < 0.05.

### Transcription factors identification

Cistrome DB Toolkit database (35) serves as a website tool adopted to obtain transcription factors (TFs) that might regulate genes. The Cistrome DB Toolkit was utilized to predict which TFs are most likely to contribute to increasing TAP1 expression in ccRCC. Then GEPIA2.0 was used to verify the relation between TAP1 and the predicted TFs.

### Stromal score, immune score, and estimate score based on TAP1 expression

ESTIMATE (https://bioinformatics.mdanderson.org/publicsoftware/estimate/) served as an algorithm applying sequencing profiles to visualize the extent of immune infiltration in a tumor microenvironment, including stromal and immune cells. STROMAL score, IMMUNE score, and ESTIMATE score were used to describe the immune infiltration. Then, the profiles of ESTIMATE scores and TAP1 were integrated to calculate the correlations. The results were considered statistically significant when p < 0.05.

### TAP1 DNA and RNA methylation profile in pan-cancer

GSCA was adopted to compare the different methylation levels of TAP1 between tumor and normal samples, spearman correlation between TAP1 mRNA expression and methylation, in different cancer types. The pan-cancer data set: TCGA, TARGET, GTEx were downloaded from UCSC (https://xenabrowser.net/) database, and the expression data of TAP1 gene and 44 marker genes modified by m1A (10), m5C (13), m6A (21) in each sample were further extracted. All the normal samples were filtered and with the transformation carried out log2 (x+0.001) on each expression value.

### CancerSEA

CancerSEA (http://biocc.hrbmu.edu.cn/CancerSEA/home.jsp) is a database for analyzing distinct functional states of different cancer cells at the single-cell level (36), where 14 functional states are involved, referring to angiogenesis, apoptosis, cell cycle, differentiation, DNA damage, DNA repair, Epithelial-Mesenchymal Transition, hypoxia, inflammation, invasion, metastasis, proliferation, quiescence and stemness. This database was adopted to analyze TAP1 function in ccRCC states with cancer single cell data.

### Drug sensitivity analysis based on CellMiner database

CellMiner (37, 38) provides an integration of molecular and pharmacological data from NCI-60 cancer cell lines. The Spearman’s test was performed for correlation analysis, with p <0.05 utilized as a screening condition.

### Immunohistochemistry

Immunohistochemistry (IHC) staining was conducted to measure the protein expression of TAP1 in 4 pairs of ccRCC tissues and adjacent normal renal tissues collected from Fudan University Shanghai Cancer Center. After deparaffinization and hydration, the slides were placed in AR9 (FreeThinking, NanJing, China) buffer for antigen retrieval at 95°C for 15 min, then blocked with goat serum for 20 min at room temperature. Slides were incubated with anti-TAP1 (11114-1-AP, Proteintech, China, diluted 1:200) at 4°C overnight and then with tritC-conjugated anti-rabbit immunoglobulin (ZSGB-Bio, PV-6004, China) at room temperature for 60 min. All the staining was carried out on the IHC/ISH System (BenchMark GX, Roche, USA) with the manufacturer’s instruction followed. All sections were assessed independently by two experienced pathologists. The immunoreactivity was defined by the brown in the cytoplasm of tumor cells.

### Cell culture

All cell lines in this study were provided by the National Collection of Authenticated Cell Cultures of China (No. TCHu186). The 786-O and 769-P cells were cultured in culture medium RPMI-1640 (GIBCO, Shanghai, China), supplemented with 10% fetal bovine serum (GIBCO, Shanghai, China). Cells were incubated in a humidified atmosphere incubator of 5% CO_2_ at 37°C.

### Cell transfection

Cells were transfected with siRNA according to the protocol of Lipofectamine 2000 reagent (Thermo Fisher Scientific, Waltham, MA, USA) in a 100 mm plate, as previously mentioned (39). The transfection dose for each well was 10 μl of siRNA1 (sequences: CGGGAUCUAUAACAACACCAU), siRNA2 (sequences: CCGUGUGUACUUAUCCUGGAU), or non-targeting siRNA (negative control) at a concentration of 50 nmol/L after incubated with Opti-MEM for 20 min. Transfected cells were harvested for at least 48 h after transfection for further experimental analysis.

### Protein isolation and western blot

Proteins were extracted from 769P and 786O cells using Western and IP lysis buffer (Beyotime Biotechnology, Jiang Su, China), as previously mentioned (40). Samples were separated by electrophoresis on 10% SDS gel (epizyme, Shanghai, China) and then transferred to a methanol-activated polyvinylidene fluoride (PVDF) membrane (Millipore, Shanghai, China). Membranes were blocked with 5% bovine serum albumin (BSA) for 1 h at room temperature and then incubated with primary antibodies, anti-TAP1 (1:1,000, 11114-1-AP, proteintech, China), and anti-beta-Actin primary antibody (1:1,000, AC026, Abclonal, China) at 4°C for 8 hours. After washed with TBST for three times, membranes were incubated with secondary antibody goat anti-rabbit IgG conjugated with HRP (1:3,000, SA00001-2, Proteintech, China) at room temperature for 60 min. After three washes with TBST for each 10 mins, the bands were visualized using ECL-plus™ western blotting chemiluminescence kits (BD Biosciences, NJ, USA). Image J was used to analyze strip grayscale, and Actin was used as a reference.

### Total RNA extraction and real time PCR

Total RNA was extracted from human ccRCC and patient-matched normal tissue samples preserved in RNAlater using TRIzol^®^ reagent (Invitrogen Life Technologies, Waltham, MA, USA) and then converted to cDNA using MasterMix (TaKaRa, Otsu, Japan), as previously mentioned (40). The TAP1 mRNA levels were measured by quantitative real-time PCR using the ABI Prism 7900 sequence detection system (Applied Biosystems, Foster City, CA, USA), with actin as an internal reference gene. Each reaction was performed in triplicate. The PCR primer sequences used for TAP1 are listed as follows: forward, 5′-TGCCCCGCATATTCTCCCT-3′ and reverse, 5′- CACCTGCGTTTTCGCTCTTG-3′. The TAP1 mRNA expression was represented as ΔCt = Ct_(TAP1)_ - Ct_(β-actin)_.

### Wound healing

The transfected cells were seeded into 6-well plates (Corning, Su Zhou, China) and cultured until almost overgrowth. Then, a wound was created by scratching the cells using 100-μL pipette tips (Pullen, Shanghai, China), as previously mentioned (41). Cell migration was evaluated by measuring the gap width in multiple fields immediately and after 18 h.

### Transwell assay

Cell migration capacity was determined using Transwell chambers (Corning, Su Zhou, China), as previously mentioned (41). A total of 20000 cells were plated in the top of the Transwell filter with a 200 μL medium without fetal bovine serum. The lower compartment was filled with an 800 μL culture medium with 10% fetal bovine serum. After incubation for 24 hours, the migrated cells were stained using crystal violet and counted using Image J.

### Statistical analysis

Statistical analysis was performed on GraphPad Prism 9.0.1 software (GraphPad, Bethesda, MD, USA). Student’s t-test was performed to evaluate the difference between two groups. Log-rank test was performed for survival analysis. Spearman’s correlation coefficient was adopted to examine relationships between TAP1 and immune parameters. P < 0.05 was considered statistically significant.

## Results

### TAP1 expression is elevated in KIRC patients, indicating a worse prognosis

**Figure 1:**
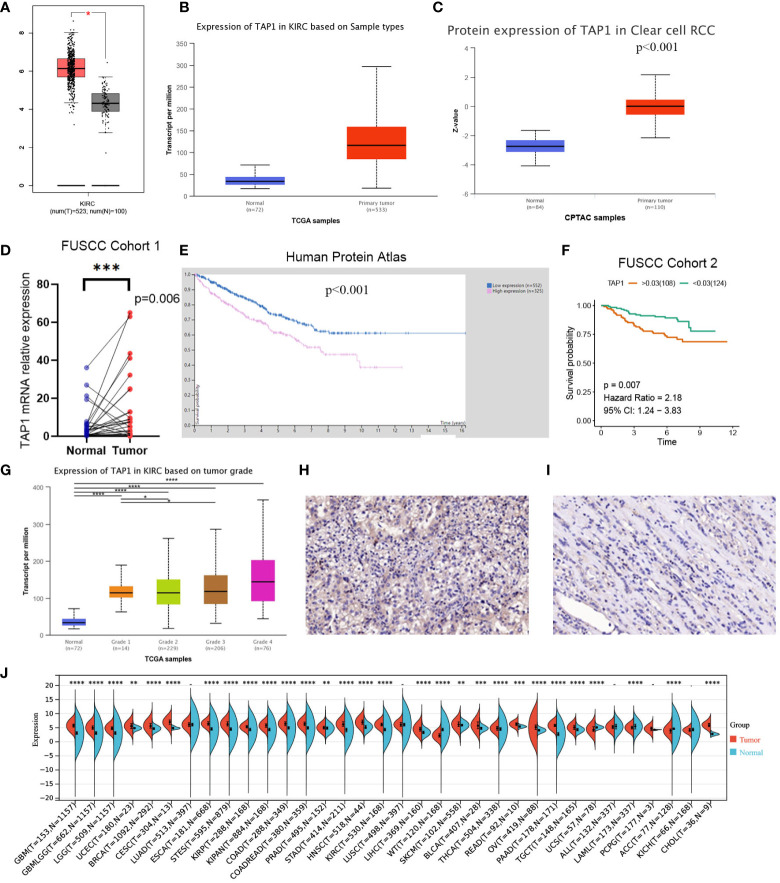
Transporter associated with antigen processing 1(TAP1) expression profiles in human normal and clear cell renal cell carcinoma (ccRCC) tissues. **(A, B)** TAP1 has a higher mRNA expression level in cancer than para-cancer tissues based on the Cancer Genome Atlas (TCGA) database. **(C)** TAP1 has a higher protein expression level in cancer than para-cancer tissues based on Clinical Proteomic Tumor Analysis Consortium (CPTAC) database. **(D)** TAP1 has a higher mRNA expression level in cancer than para-cancer tissues based on Fudan University Shanghai Cancer Center (FUSCC) cohort 1. **(E, F)** Survival curves in Human Protein Atlas (n=877, p<0.001) and FUSCC proteomic cohort (n=232, p<0.01) with significance. **(G)** TAP1 expression is positively correlated with advanced cancer (*, P < 0.05; **, P < 0.01; ***, P < 0.001; ****, P < 0.0001). **(H, I)** Immunohistochemical analysis showed a higher TAP1 expression level in ccRCC samples. **(J)** TAP1 expression levels in para-cancer tissues from the TCGA database.

**Figure 2:**
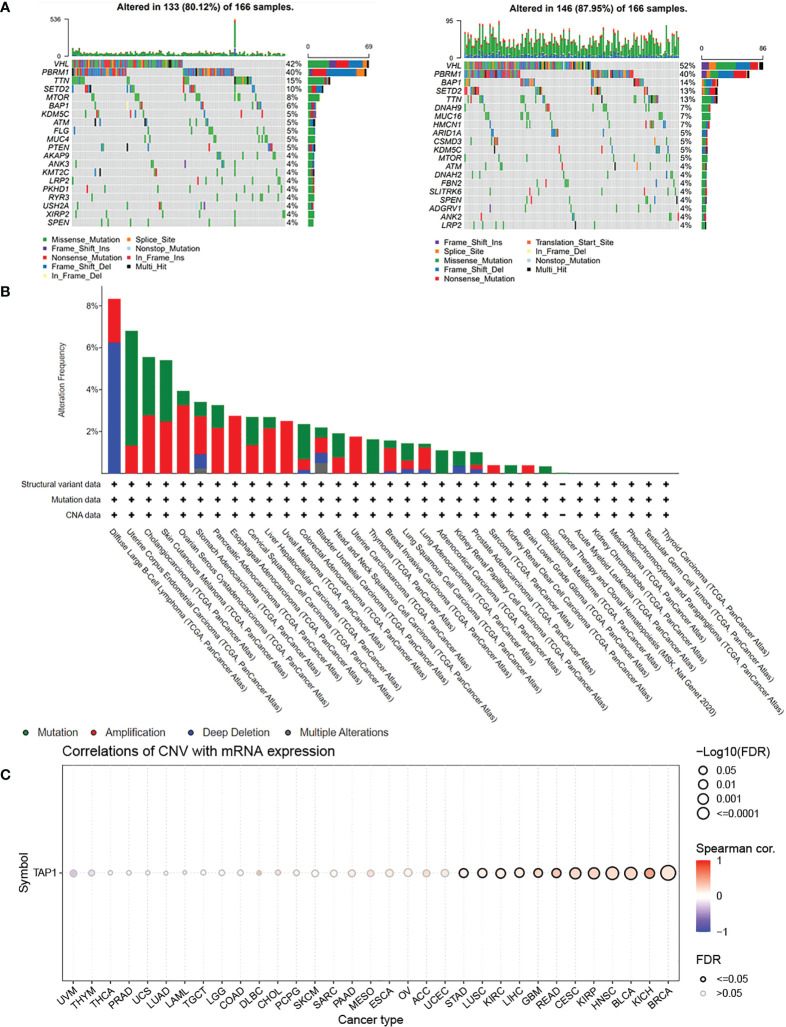
Genomic Alteration Analysis of TAP1. **(A)** Landscape of genomic alteration in low-(Left) and high-(Right) expression groups of TAP1. **(B)** Alteration Frequency of TAP1 in para-cancer indicates TAP1 is conservative in Kidney renal clear cell carcinoma (KIRC). **(C)** TAP1 mRNA expression is highly related to copy number variations (CNV) in KIRC.(p < 0.05).

TAP1 expression in ccRCC was analyzed by adopting several databases. In GEPIA, tumor tissues exhibited a higher mRNA expression level in comparison to normal tissues (**Figure 1A**, p<0.05), which was consistently shown in UALCAN (**Figure 1B**, p<0.001). As for protein expression levels, cancer tissues displayed a higher KIRC in comparison to normal tissues (**Figure 1C**, p<0.001). To determine the TAP1 expression between normal tissues and Chinese ccRCC tissues, the qPCR using ccRCC samples from our center was carried out. In the Chinese population, ccRCC patients also showed an elevated TAP1 expression, paired Student’s t-test was performed for statistical analysis (P < 0.001 significance). (**Figure 1D**, p=0.006). Based on tumor grade, TAP1 expression was positively correlated with advanced cancer **(** **Figure 1G** **)**. The immunohistochemical results of ccRCC from our center also indicated that TAP1 is highly expressed in ccRCC tissues **(** **Figures 1H, I** **)**. In order to figure out the prognostic value of TAP1, the TCGA proteomics database was used for analysis. The results of survival analysis demonstrated that higher expression was associated with a worse prognosis (**Figure 1E**, p<0.001), as consistently indicated by the proteomics database from Fudan University Shanghai Cancer Center (FUSCC) (**Figure 1F**, p=0.007). Then we aimed to figure out TAP1 expression in pan-cancer (**Figure 1J**). The expression of TAP1 was increased in 25 cancers, including Glioblastoma Multiforme (GBM), Brain Lower Grade Glioma (LGG), Breast invasive carcinoma (BRCA), Kidney renal papillary cell carcinoma (KIRP), Colon adenocarcinoma (COAD), Prostate adenocarcinoma (PRAD), KIRC, and so on, while decreased in Adrenocortical carcinoma (ACC). We then investigated the changes in TAP1 at the genomic level. The landscape of genomic alteration in low- and high- expression groups is shown in **Figure 2A**. The gene mutation analysis at the pan-cancer level suggested the relatively lower mutation of TAP1 at the genome level of patients with KIRC (**Figure 2B**). Moreover, in ccRCC patients, TAP1 mRNA expression exhibited a positive tendency to copy number variations (CNV) (**Figure 2C**, p<0.05).

### Low expression of TAP1 inhibits migration in ccRCC cell lines

**Figure 3:**
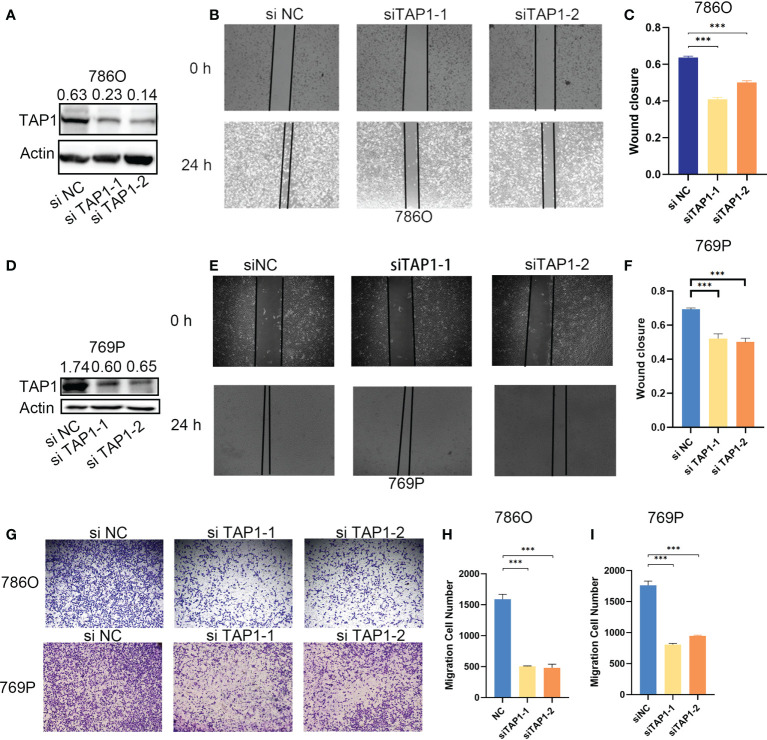
TAP1 promotes migration of human ccRCC cells. **(A, D)** TAP1 was decreased after transfected with TAP1-siRNA in 786O(A) and 769P(D). Representative images of three independent reproducible experiments are shown. **(B, C, E, F)** Wound healing assays detected the effects of inhibiting TAP1 expression on cell migration. Representative images of three independent reproducible experiments are shown. Scale bar: 200 μm (***, P < 0.001). **(G–I)** Transwell assays detected the effects of inhibiting TAP1 expression on cell migration. Representative images of three independent reproducible experiments are shown. Scale bar: 200 μm. Data are shown as mean ± SD in **(C, F, H, I)**. P values are derived from t test. (***, P < 0.001).

In order to explore the function of TAP1 in ccRCC *in vitro*, we knocked down the TAP1 in ccRCC cell lines, 786O and 769P, followed by experimental validation. According to the western blot, low TAP1 expression displayed significance after transfected with siRNA (**Figures 3A, D** **)**. Then, scratches were created of equal width in a six-well plate confluent with cells, which were photographed under the microscope with the width and wound healing area measured. After 12 hours, the same scratches were photographed under the microscope accompanied with the measurement of wound healing area (**Figures 3B, C, E, F**, p<0.001). It was indicated that the lower TAP1 expression contributed to slowing the speed of wound healing. Afterwards, we seeded 20,000 cells in Transwell chambers, where the tumor cells tended to migrate to places with high levels of nutrients. Cells with decreased TAP1 expression led to fewer cell numbers through the membrane (**Figures 3G, H, I**, p<0.001). These results demonstrated that low expression of TAP1 resulted in the inhibition of migration in ccRCC cell lines. TAP1 may play a role in ccRCC distant metastasis.

### TAP1 is associated with immune infiltration and immune checkpoint genes

Considering few studies of TAP1 focused on caner immunity, especially in RCC tumor immunity, herein we performed a comprehensive analysis covering correlation analysis of TAP1 expression and immune-regulated genes, immune checkpoint inhibitor genes, and immune cell infiltration degree in pan-cancer levels.

**Figure 4:**
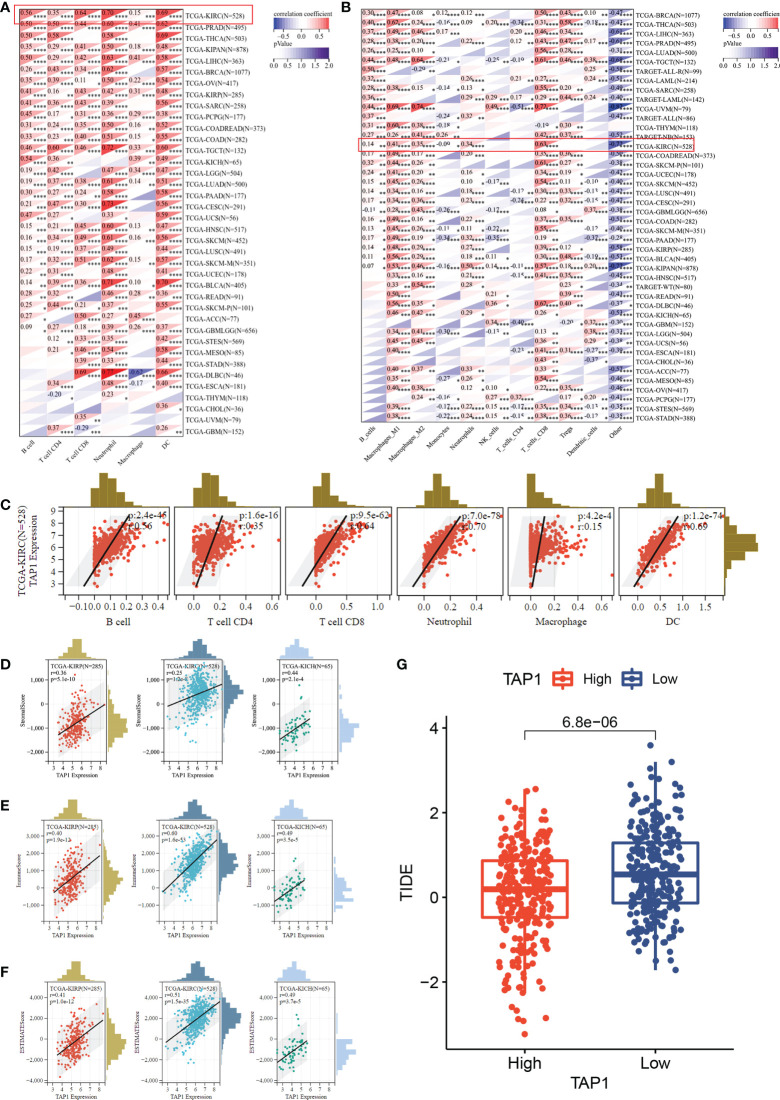
TAP1 expression is positively correlated with immune cell infiltration *via* multiple algorithms. **(A)** The landscape of TAP1 correlating with various immune infiltrates in pan-cancer with The tumor immune estimation resource (TIMER). **(B)** The landscape of TAP1 correlating with various immune infiltrates in pan-cancer with QUANTISEQ. **(C)** Scatter plot showed relationship between immune cells and TAP1 expression in KIRC, including B cells (cor. = 0.56), CD4+ T cells (cor. = 0.35), CD8+ T cells (cor. = 0.64), Neutrophil (cor. = 0.70), Macrophage (cor. = 0.15), dendritic cell (DC) (cor. = 0.69). **(D-F)** Immune infiltration score of TAP1 in KIRC, Kidney renal papillary cell carcinoma (KIRP), Kidney Chromophobe (KICH). **D**, Stromal Score, **E**, Immune Score, F, ESTIMATE Score. **(G)** High level of TAP1 expression indicates better immune checkpoint inhibitor response based on Tumor Immune Dysfunction and Exclusion (TIDE) (p < 0.0001). *, P < 0.05; **, P < 0.01; ***, P < 0.001; ****, P < 0.0001.

Multiple algorithms were adopted to identify cancer immune infiltration. In TIMER database, TAP1 exhibited a positive association with immune cells infiltration in KIRC, covering B cells, CD4+ T cells, CD8+ T cells, Neutrophil cells, Macrophage cells, and dendritic cells (**Figure 4A**), as were displayed in a form of scatter plots (**Figure 4C**). According to the quanTIseq database, TAP1 additionally displayed a positive correlation with B cells, M1-Macrophages, M2-Macrophages, Neutrophils, CD8+ T Cells, while an inverse relation with Monocytes (**Figure 4B**).

The ESTIMATE-STROMAL-IMMUNE score is required for the accurate prognostic markers for the prediction of clinical outcomes. We analyzed the correlation of TAP1 expression with ESTIMATE, stromal, and immune scores in KIRC, KIRP, and KICH. As indicated in **Figures 4D-F**, TAP1 had a positive correlation with ESTIMATE, stromal, and immune scores, suggesting more immune and stromal cells infiltration in high-TAP1 expression samples. Tumor Immune Dysfunction and Exclusion (TIDE) serves as a computational framework for predicting results about immunotherapy (42, 43). It was found that high TAP1 expression produced a better response to immune checkpoint inhibitors (**Figure 4G**, p< 0.0001).

**Figure 5:**
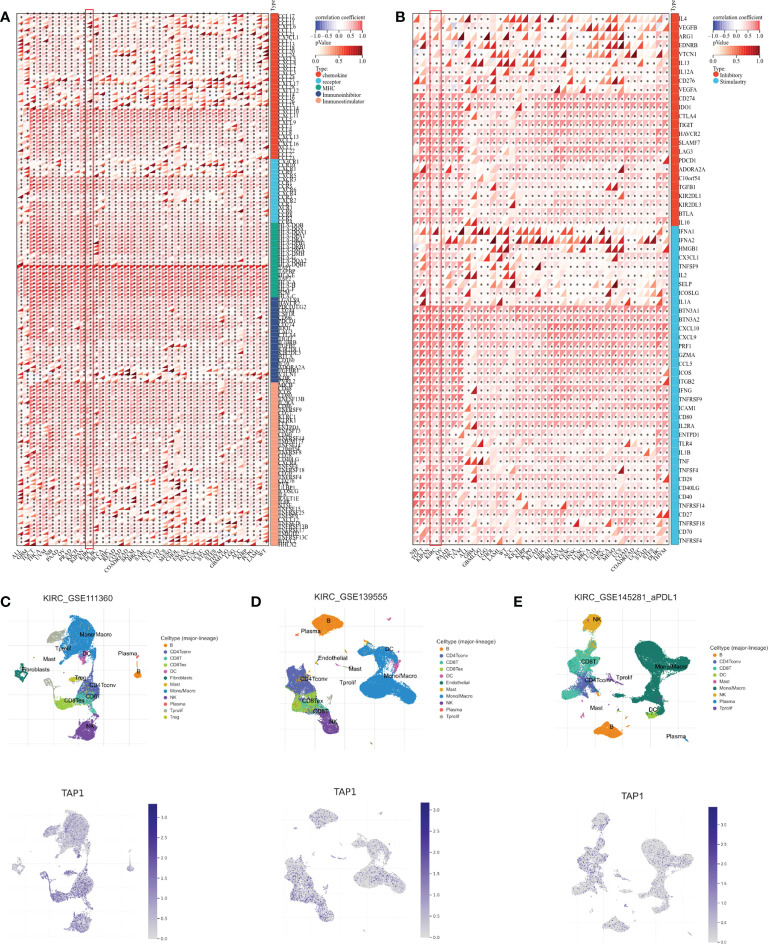
Association between TAP1 and tumor immunity in pan-cancer. **(A)** Landscape of immune regulator genes based on TAP1 expression 39 cancer types, including chemokine, receptor, major histocompatibility complex (MHC), Immuno-inhibitor, Immuno-stimulator. **(B)** Landscape of immune checkpoint-related genes based on TAP1 expression in 39 cancer types, including inhibitory and stimulatory. **(C-E)** Single-cell sequencing datasets GSE111360, GSE139555, and GSE145281 suggested localization of TAP1 mainly involve monocytes/macrophages, CD4+ and CD8+ T cells, B cells, mast cells, dendrite cells in ccRCC. *, P < 0.05; **, P < 0.01; ***, P < 0.001; ****, P < 0.0001.

In addition to the tumor immune microenvironment, the profile on some immune regulator genes was also conducted. The expression showed a significantly positive correlation with chemokine, chemokine receptors, MHC, and immuno-inhibitor and immuno-stimulator genes in multiple cancer types, covering ALL, GBM, Testicular Germ Cell Tumors (TGCT), Thyroid carcinoma (THCA), Uveal Melanoma (UVM), Pancreatic adenocarcinoma (PAAD), Ovarian serous cystadenocarcinoma (OV), PRAD, KICH, Lymphoid Neoplasm Diffuse Large B-cell Lymphoma (DLBC), Breast invasive carcinoma (BLCA), Liver hepatocellular carcinoma (LIHC), Rectum adenocarcinoma (READ), Colon adenocarcinoma (COAD), Rectum adenocarcinoma (READ), Skin Cutaneous Melanoma (SKCM), Breast invasive carcinoma (BRCA), Sarcoma (SARC), Cervical squamous cell carcinoma and endocervical adenocarcinoma (CESC), Lung adenocarcinoma (LUAD), Uterine Carcinosarcoma (UCS), Mesothelioma (MESO), Cholangiocarcinoma (CHOL), Esophageal carcinoma (ESCA), Head and Neck squamous cell carcinoma (HNSC), Lung squamous cell carcinoma (LUSC), Uterine Corpus Endometrial Carcinoma (UCEC), Stomach adenocarcinoma (STAD), Stomach and Esophageal carcinoma (STES), Thymoma (THYM), Glioblastoma Multiforme Brain Lower Grade Glioma (GBMLGG), LGG, ACC, KIRP, Pheochromocytoma and Paraganglioma (PCPG), Acute Myeloid Leukemia (LAML) (**Figure 5A**). Correspondingly, TAP1 exhibited a positive correlation with immune stimulus genes and inhibitory genes in cancers mentioned above (**Figure 5B**). Finally, it was expected to figure out TAP1 abundance in different kinds of cells in ccRCC tumors using single-cell databases. TAP1 was found widely distributed in various types of cells, involving monocytes/macrophages, CD4+ and CD8+ T cells, B cells, mast cells, dendrite cells, and so on. (**Figures 5C-E**)

### Identification of factors regulating TAP1

**Figure 6:**
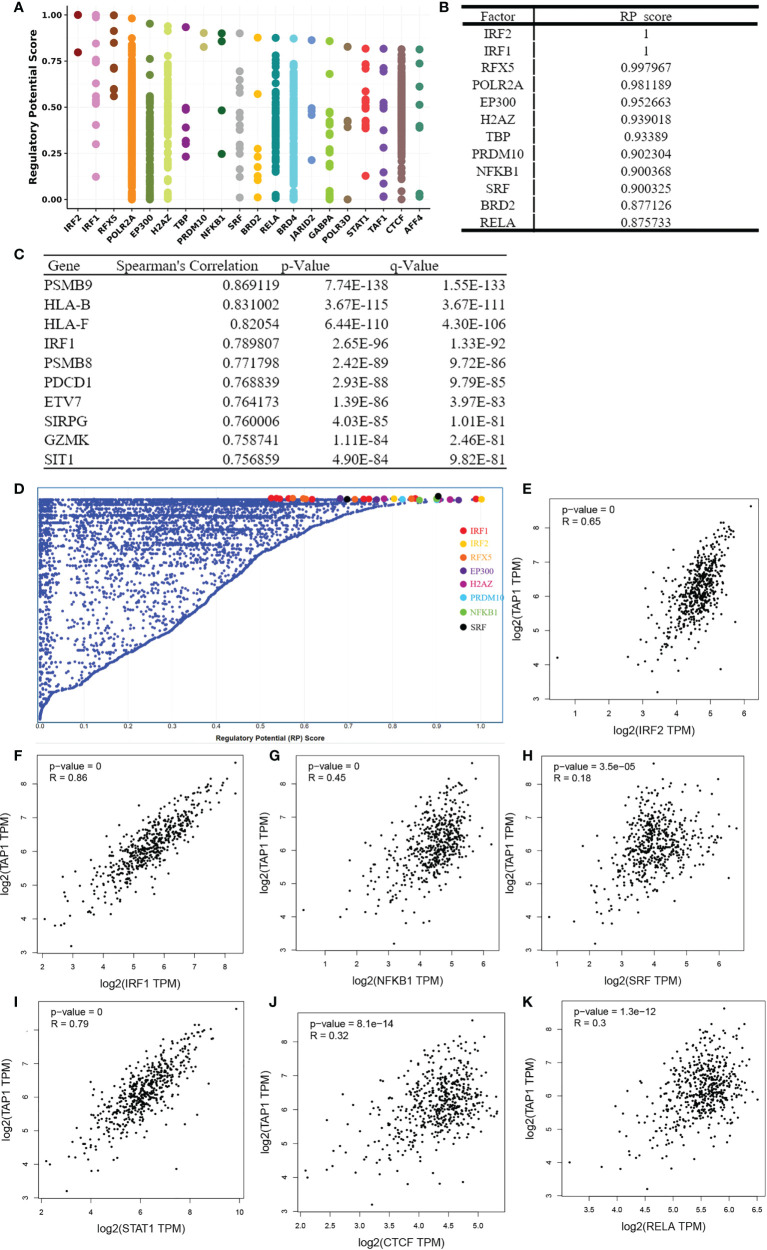
Identification of TAP1-related Transcription factors(TFs). **(A, D)** TAP1 was regulated by those 20 most likely TFs in human cancers. **(B)** Top 12 transcription factors based on Regulatory Potential (RP) Scores. **(C)** Top 10 genes correlated with TAP1 obtained from cBioportal database. **(E-K)** There’s a connection between TAP1 mRNA expression and these transcriptional factors, such as IRF2 (cor. = 0.65), IRF1 (cor. = 0.86), NFKB1 (cor. = 0.45), SRF (cor. = 0.18), STAT1 (cor. = 0.79), CTCF (cor. = 0.32), and RELA (cor. = 0.3) in KIRC based on Gene Expression Profiling Interactive Analysis (GEPIA) database.

To identify factors of a potential molecular network that affect TAP1 expression, transcription factors that might regulate TAP1 gene transcription were examined using the Cistrome DB Toolkit. We referred to the regulatory potential score to predict potential TFs combing with TAP1 promoter. As indicated in **Figures 6A, B, D**, the top 12 TFs were listed, that were, IRF2, IRF1, RFX5, POLR2A, EP300, H2AZ, TBP, PRDM10, NFKB1, SRF, BRD2, RELA, which involved in some critical signaling pathways. Then, we figured out some genes related with TAP1 expression using cBioportal database, with 10 of the most significant genes listed (**Figure 6C**), among which were some TFs. In order to further determine the existence of significant correlation in all these TFs with TAP1, we attempted to reveal the potential relationship *via* GEPIA database (**Figures 6E-K**). Most transcription factors exhibited a significant correlation with TAP1, referring to IRF2 (cor. = 0.65), IRF1 (cor. = 0.86), NFKB1 (cor. = 0.45), SRF (cor. = 0.18), STAT1 (cor. = 0.79), CTCF (cor. = 0.32), and RELA (cor. = 0.3).

DNA and RNA methylation modifications are considered classic epigenetics biomarkers. The former plays a role in regulating the dynamic transition between euchromatin and heterochromatin, which changes chromosome accessibility. The latter is involved in the whole process of RNA production, covering RNA splicing, decay, and distribution outside and inside nuclear. As shown in **Supplementary Figure 1A**, the correlation of methylation with TAP1 expression exists in BRCA, KIRP, COAD, LIHC, SARC, THCA, and CHOL, without a correlation observed between methylation and TAP1. However, the correlation between TAP1 and RNA modification-associated enzyme was observed in most of cancers, indicating an engagement of RNA methylation in post-translation regulation **(** **Supplementary Figure 1B** **)**.

### Identify the biological function of TAP1

**Figure 7:**
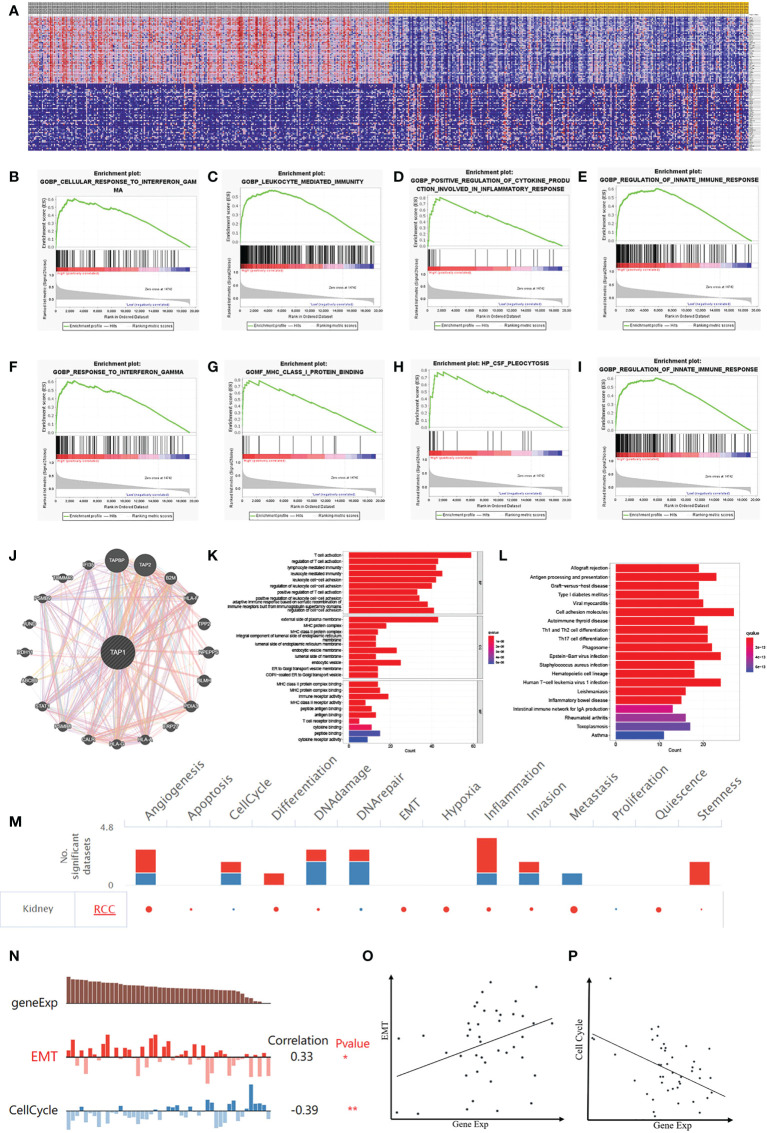
Potential Biological Functions of TAP1 of ccRCC. **(A)** Heatmap shows Differentially Expressed Genes (DEGs) between low- and high-TAP1 expression groups obtained from TCGA database. **(B-I)** Gene Set Enrichment Analysis (GSEA) analysis indicated high expression of TAP1 is enriched in functional states including cellular response to interferon-gamma **(B)**, leukocyte-mediated immunity **(C)**, positive regulation of cytokine production involved in an inflammatory response **(D)**, regulation of innate immune response **(E)**, and response to interferon-gamma **(F)**, MHC class I protein binding **(G)**, CSF pleocytosis **(H)**, regulation of innate immune response **(I)**. **(J)** Protein‐protein interaction (PPI) network showed factors significantly coregulated with TAP1. **(K)** Gene Ontology (GO) function analysis of DEGs. The top group represents biological process (BP) group, the middle group represents cellular component (CC) group and the bottom group represents molecular function (MF) group. **(L)** Functional enrichment analysis of DEGs based on the Kyoto Encyclopedia of Genes and Genomes (KEGG) database, including allograft rejection, antigen processing and presentation, Graft-versus-host disease, Type I diabetes mellitus viral myocarditis, and so on. **(M)** The functional state of TAP1 in ccRCC based on CancerSEA. The red plots indicated that TAP1 is positively correlated with the functional state while the blue plots indicated that TAP1 was negatively correlated with the functional state identified by CancerSEA. **(N)** Single-cell analysis indicated that TAP1 is primarily involved in epithelial–mesenchymal transition (EMT) and Cell Cycle in ccRCC. **(O)** Correlation between TAP1 expression and EMT (Cor=0.33). **(P)** Correlation between TAP1 expression and Cell Cycle (Cor= -0.39).

GSEA (**Figure 7**) was performed for clarifying the biological role of TAP1. In order to illustrate the potential downstream pathway, a heatmap was plotted to distinguish the top 100 differential genes (positive and negative), with the landscape depicted in **Figure 7A**. These genes were further analyzed by performing Gene Set Enrichment Analysis (GSEA). The processes most significantly associated with TAP1 referred to a cellular response to interferon-gamma, leukocyte-mediated immunity, positive regulation of cytokine production involved in an inflammatory response, regulation of innate immune response, and response to interferon-gamma, MHC class I protein binding, CSF pleocytosis, regulation of innate immune response (**Figures 7B-I**). PPI network of Differentially Expressed Genes (DEGs) was established (**Figure 7J**). Gene Ontology (GO) functional analysis revealed that the DEGs were mainly enriched by T cell activation and lymphocyte-mediated immunity in the biological process (BP) group. The external side of the plasma membrane lied the most enriched term in the cellular component (CC) group. In the molecular function (MF) group, the DEGs were mainly enriched in MHC protein complex binding (**Figure 7K**). The Kyoto Encyclopedia of Genes and Genomes (KEGG) demonstrated the hub genes mainly enriched in cell adhesion molecules, allograft rejection, antigen processing and presentation (**Figure 7L**).

Currently the TAP1 is closely related to immune microenvironment, which might exert an influence depending on immune cells, or tumor microenvironment. To illustrate the effect, a single cell database “CancerSEA” was adopted to explore biological function **(** **Figure 7M** **)**. Functional relevance analysis revealed the positive correlation of TAP1 expression with metastasis **(** **Figure 7O**, cor. = 0.33); and inverse correlation with Cell Cycle in RCC (**Figure 7P**, cor.= -0.39).

Given the prominent role of TAP1 in interferon signaling pathway, we further analyzed the expression of IRF1, STAT1, STAT2, and JAK1 between the low- and high-TAP1 groups, finding the increasing expression of them in the high-TAP1 group (**Supplementary Figures 2A-D**). Meanwhile, we acquired the IFN response signature expression, demonstrating that TAP1 level was positively associated with expression of IFN response signature genes in RCC population (**Supplementary Figure 2E**) (44).

### Potential chemo drugs for TAP1 determined ccRCC progress

**Figure 8:**
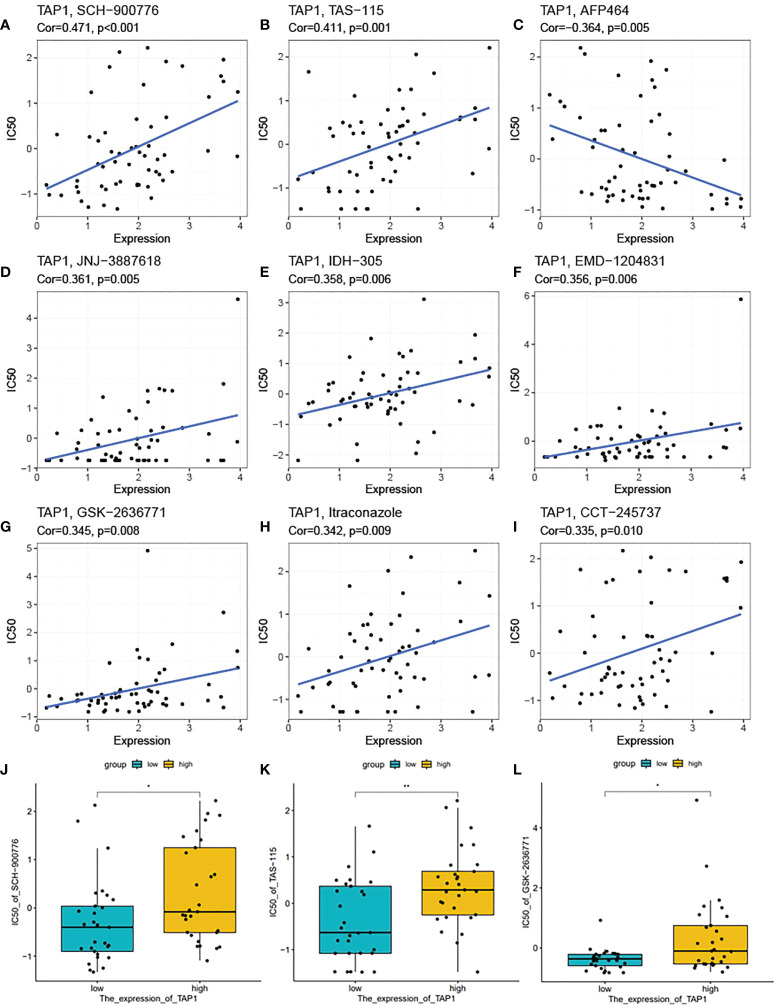
TAP1 impacted potential effect of chemotherapy drugs. **(A-I)** Correlation of TAP1 expression and half maximal inhibitory concentration (IC50) of different drugs obtained from the CellMiner database, including SCH-900776 (**A,** Cor=0.471, p<0.001), TAS-115 (**B**, Cor=0.411, p=0.001), AFP464 (**C**, Cor=-0.364, p=0.005), JNJ-3887618 (**D**, Cor=0.361, p=0.005), IDH-305 (**E**, Cor=0.358, p=0.006), EMD-1204831 (**F**, Cor=0.356, p=0.006), GSK-2636771 (G, Cor=0.345, p=0.008), Itraconzole (**H**, Cor=0.342, p=0.009), CCT-245737 (**I**, Cor=0.335, p=0.010). **(J-L)** Different IC50 of medicines based on the expression of TAP1 downloaded from the Cancer Cell Line Encyclopedia (CCLE) database (**J**, SCH-900776, **K**, TAS-115, **L**, GSK-2636771). *, P < 0.05; **, P < 0.01.

In recent days, precise therapy highlights the increasing role for patients (45–48). We attempted to identity the potentially effective chemo-drugs to inhibit TAP1-regulated oncogenic process. It was demonstrated that TAP1 expression was positive with the half maximal inhibitory concentration (IC50) of SCH-900776 (cor. = 0.471), TAS-115 (cor. = 0.411), JNJ-3887618 (cor. = 0.361), IDH-305 (cor. = 0.358), EMD-1204831 (cor. = 0.356), GSK-2636771 (cor. = 0.345), Itraconazole (cor. = 0.342), CCT-245737 (cor. = 0.335), suggesting the availability of these chemo-drugs for the treatment with low level of TAP1, while AFP464 (cor. = 0.364) might be more suitable for higher TAP1 expression (**Figures 8A-I**). Another database was also enrolled and presented that SCH-900776, TAS-115, GSK-2636771 are suitable for patients with lower TAP1 (**Figures 8J-L**).

## Discussion

CcRCC is considered a kind of cancer with high-level immune cell infiltration (49), for which the immunotherapy represented by PD1 has a rather promising prospect in the treatment. However, resulting from the huge heterogeneity of the immune system (50), the efficacy of immunotherapy represented by PD1 is reported not as good as expected (51). Therefore, further study of the formation mechanism of immune microenvironment will contribute to providing the source understanding on the heterogeneity of tumor immune microenvironment.

TAP1 serves as a transporter engaged in antigen-processing of MHC-I for recognition by CD8+ T lymphocytes. Physiologically, the TAP1/TAP2 peptide transporter complex brings antigen peptide substrates into the endoplasmic reticulum, where they are processed and loaded onto MHC-I for exported to the cell surface *via* the Golgi apparatus (52). In theory, the downregulation of the expression of TAP1 can lead to the decreased MHC-I complexes on the cell membrane, so that tumor cells can escape cytotoxic T cell recognition (53). It also has been reported that TAP1 mutation can produce an effect on the MHC-I function of tumor surveillance (54, 55). A previous study proposed TAP1 to be adopted as cancer treatment *via* immunotherapy considering its importance in the peptide MHC-I complex and as the role of elevating the immune response (56, 57). However, the mechanism of TAP1 affecting tumor biological behavior varies among different tumors. In early-stage breast cancer, the levels of TAP1 expression reached low to negative (16), which may contribute to evading the attack of the immune system during tumor formation. However, as the tumor progresses, the expression level of TAP1 is increased. In colon rectal cancer (CRC), a low expression of TAP1 was also obviously associated with poor prognosis in patients with CRC (14). In ovarian cancer, overexpression of TAP1 in OC patients leads to a poor prognosis. The high expression of TAP1 promotes tumor migration and metastasis *in vivo* and *in vitro (* 58). Our study confirmed the increased mRNA levels of TAP1 in ccRCC, both in the TCGA cohort and in the FUSCC cohort, as well as the increased protein expression in the proteomic study. And in ccRCC, the high expression of TAP1 indicated a poor prognosis, which is consistent with previous studies (59). On the other hand, the TAP1 conservation, that is the less genomic alteration, in ccRCC patients, also indicates that wild-type TAP1 may play a critical role. The results of experiments *in vitro* demonstrated that the high expression of TAP1 contributes to tumor migration, suggesting its role in metastasis.

Considering the role of TAP1 in the process of antigen presentation, it potentially has a close relation to tumor immunity. We analyzed the relationship between the high expression of TAP1 and tumor immune infiltration. Despite the different algorithms, both reveal the association of high TAP1 expression with a variety of immune cell infiltration, especially CD8+T cells. Our results indicated a positive correlation existing between the high expression of TAP1 and the abundance of infiltration in both immune cells and stromal cells. It can be concluded that the down-regulation of TAP1 expression in early tumors contributes to preventing early tumor antigens from recognized by the immune system, and finally achieving the immune escape. In colorectal cancer, Kasajima et al. have demonstrated the relation of the down-regulation of TAP1 expression to the loss of inflammatory response (25). However, this down-regulation does not mean the deficiency of tumor antigen presentation function. Leone et al. have indicated that TAP1 down-regulation might lead to the presentation of empty HLA molecules (60), which means that tumor cells could evade recognition from NK cells by expressing HLA molecules on their surface. Afterwards we analyzed some immunomodulatory genes in ccRCC, finding that the high expression of TAP1 was positively correlated with many immunomodulatory genes. However, due to the positive correlation of TAP1 with most genes, which refer to immune stimulus genes and immunosuppressive genes, so the overall effect is not significantly clear. Therefore, due to the different genetic backgrounds, the overall effect of TAP1 in different cancer species is varying.

Surprisingly, in the analysis of TCGA proteome database, the expression of TAP1 in early ccRCC (stage 1 and 2) was shown increased in line with the increase of tumor stage. This illustrates the involved of TAP1 in other mechanisms in the occurrence and development of ccRCC in addition to antigen presentation.

Then we expected to explore the primary factors affecting the expression of TAP1, in which the transcription factor-mediated transcriptional regulation lies the most common point of view (61). We analyzed the transcription factors possibly binding to the TAP1 promoter region by Cistrome DB Kit, screening out the IRF2, IRF1, NFKB1, RELA, STAT1, which were then evaluated for the correlation with TAP1 expression. It was revealed that the expression of these transcription factors was positively correlated with the expression of TAP1 in ccRCC. Notably, IRF1 and STAT1 associate TAP1 with the interferon pathway. In HeLa cells, IFN-γ–activated Stat1α/Stat1α binds to a GAS in the promoter of TAP1, so as to mediate the delayed response of HLA class I promoter (62–64).

Then we examined the epigenetic regulation of TAP1, revealing that there exhibited no significant correlation of the high expression of TAP1 with DNA methylation in ccRCC. On the contrary, a good correlation was shown between the expression of TAP1 and the enzymes related to RNA modification, indicating that the expression of TAP1 is mainly determined by the post-transcriptional regulation mediated by RNA methylation. This is distinguished to previous studies in many cancers, which, however, are mainly focused on the low expression of TAP1 in the early stage of tumorigenesis, suggesting the relation of low expression of TAP1 to the high level of DNA methylation (65). Studies on TAP1 and epigenetic modification are also rather limited. Sultan M et al. have suggested that epigenetic silencing of TAP1 in Aldefluor + breast cancer stem cells contributes to the enhanced survival under immune pressure (66).

In order to identify the downstream pathways involved in TAP1, we analyzed the molecule by GSEA. The enrichment of GSEA in immune-related pathways further proved the relationship between TAP1 and tumor immunity, followed by the analysis of the genes related to the interferon pathway. It was revealed that TAP1 was positively correlated with the interferon response gene set.

With the objective to investigate the physiological function of TAP1 in tumors, single-cell database CancerSEA was utilized here for prediction. We found a negative correlation of the high expression of TAP1 with the cell cycle, which may be due to the activation of interferon pathway induced by the high expression of TAP1. However, no significant difference was shown in the cell experiment *in vitro*, possibly resulting from the requirement of CD8+T cells to participate in TAP1’s influence on cell proliferation through immunological pathway, while the functional experiment *in vitro* fails to meet this material basis.

Considering that the expression of TAP1 is associated with drug resistance in increasing types of cancers (67), we chose the CellMiner database to analyze the relationship between TAP1 expression and drugs. It can be summarized that the IC50 increases in line with the increase of TAP1 expression in most drugs, which suggests the potential drug resistance under the high expression of TAP1. However, for AFP464, they exhibited a positive correlation, suggesting that the high expression of TAP1 may elicit more sensitivity of cells to AFP464. One common source by which cancer cells achieve chemoresistance is increasing drug efflux from cancer cells mediated by TAP1 (68), which leads to a decrease in intracellular drug accumulation and the attenuation of the drug efficiency. TAP1 displays high expression in side population (SP) cells in PDAC, and SP cells have higher chemoresistance in comparison to other tumor cells, which was considered the candidate to the cancer stem cells (69). TAP1 as the downstream target of SHH signaling enhances the drug resistance in pancreatic adenocarcinoma, providing a promising therapeutic approach for the development of more effective targeted therapies in the treatment of PDAC patients (68). According to Zhou et al (27, 70), TAP1 plays a crucial role in the hedgehog signaling in mediating chemo‐resistance in hepatocellular carcinoma.

The results of experiments *in vitro* demonstrate the contributive role of high TAP1 expression in tumor migration, suggesting its contribution in metastasis. In ovarian cancer, the expression of TAP1 is related to MEF2A, LEF1, while MEF2A can promote tumor cell metastasis by inducing epithelial-mesenchymal transformation and activating Wnt/β-catenin signal pathway (71). LEF1 can promote tumor cell metastasis by participating in epithelial–mesenchymal transition (EMT) and manipulating intracellular reactive oxygen species (ROS) (72). These results serve as a basis for further study of the mechanism of TAP1 in tumor. However, the role of TAP1 in ccRCC remains to be further studied.

## Conclusion

In summary, this study aims to uncover the unknown role of TAP1 in ccRCC. We found that TAP1 had a higher expression in ccRCC, which indicated a worse prognosis. High expression of TAP1 promtes tumor metastasis. Immune landscape characterization demonstrated the significant association of TAP1 with the tumor immune microenvironment in ccRCC. In view of the role of TAP1 in tumor drug resistance, we also predicted the drug AFA464 that may be effective against high TAP1 expression. These results identify TAP1 as a novel therapeutic target for ccRCC therapy from bench to clinic.

## Data availability statement

The raw data supporting the conclusions of this article will be made available by the authors, without undue reservation.

## Ethics statement

Ethical approval for this study was provided by the Ethics Committee of the Fudan University Shanghai Cancer Centre.

## Author contributions

The work presented here was carried out in collaboration among all authors. C-YG, D-WY, G-HS, KC and Y-YQ defined the theme of the study and discussed analysis, interpretation, and presentation. Z-DW, XT, YW and KC drafted the manuscript, analyzed the data, developed the algorithm, and explained the results. J-JW, S-QY and Y-QH participated in the collection of relevant data and helped draft the manuscript and helped to perform the statistical analysis. All authors contributed to the article and approved the submitted version.

## Funding

This work is supported by Grants from the National Key Research and Development Program of China (No.2019YFC1316005), National Natural Science Foundation of China (No.81772706, No.81802525 and No.81902568), Shanghai Science and Technology Committee (No.20ZR1413100, No.18511108000), and Shanghai Sailing Program (No.19YF1409700).

## Acknowledgments

We thank the TCGA databases for providing ccRCC gene expression profiles.

## Conflict of interest

The authors declare that the research was conducted in the absence of any commercial or financial relationships that could be construed as a potential conflict of interest.

## Publisher’s note

All claims expressed in this article are solely those of the authors and do not necessarily represent those of their affiliated organizations, or those of the publisher, the editors and the reviewers. Any product that may be evaluated in this article, or claim that may be made by its manufacturer, is not guaranteed or endorsed by the publisher.

## Supplementary material

Supplementary Figure 1 DNA and RNA methylation modification in TAP1. **(A)** TAP1 expression is inversely related to DNA methylation in KIRC. **(B)** TAP1 expression is positively correlated with RNA methylation in KIRC.

Supplementary Figure 2 TAP1 is positively correlated with interferon (IFN) signaling pathway. **(A)** IRF1 expression between low and high-TAP1 expression groups downloaded from the TCGA Database. **(B)** STAT1 expression between low and high-TAP1 expression groups downloaded from the TCGA Database. **(C)** STAT2 expression between low and high-TAP1 expression groups downloaded from the TCGA Database. **(D)** JAK1 expression between low and high-TAP1 expression groups downloaded from the TCGA Database. **(E)** Heatmap show the positive correlation of TAP1 expression with IFN response signature genes in the TCGA RNA-Seq Database of ccRCC. Statistical significance was determined by Pearson’s correlation test (*, P < 0.05; **, P < 0.01; ***, P < 0.001; ****, P < 0.0001).

## Glossary

**Table:**

RCC	renal cell carcinoma
ccRCC	clear cell renal cell carcinoma
mccRCC	metastatic clear cell renal cell carcinoma
TCGA	the Cancer Genome Atlas
FUSCC	Fudan University Shanghai Cancer Center
GEO	Gene Expression Omnibus
GSEA	gene set enrichment analysis
TME	tumor microenvironment
TAP1	Transporter associated with antigen processing 1
IFN	interferon
IRF1	Interferon Regulatory Factor 1
IRF2	Interferon Regulatory Factor 2
NF-KB1	nuclear factor kappa B subunit 1
EMT	epithelial–mesenchymal transition
ICI	immune checkpoint inhibitors
MHC I	major histocompatibility complex I
KIRC	Kidney renal clear cell carcinoma
KIRP	Kidney renal papillary cell carcinoma
KICH	Kidney Chromophobe
GEPIA	Gene Expression Profiling Interactive Analysis
HPA	Human Protein Atlas
TIMER	The tumor immune estimation resource
TIICs	tumor-infiltrating immune cells
TCGA	The Cancer Genome Atlas
TFs	Transcription factors
GBM	Glioblastoma Multiforme
LGG	Brain Lower Grade Glioma
BRCA	Breast invasive carcinoma
COAD	Colon adenocarcinoma
PRAD	Prostate adenocarcinoma
ACC	Adrenocortical carcinoma
TIDE	Tumor Immune Dysfunction and Exclusion
TGCT	Testicular Germ Cell Tumors
THCA	Thyroid carcinoma
UVM	Uveal Melanoma
COAD	Colon adenocarcinoma
READ	Rectum adenocarcinoma
PAAD	Pancreatic adenocarcinoma
OV	Ovarian serous cystadenocarcinoma
BRCA	Breast invasive carcinoma
BLCA	Breast invasive carcinoma
DLBC	Lymphoid Neoplasm Diffuse Large B-cell Lymphoma
CESC	Cervical squamous cell carcinoma and endocervical adenocarcinoma
LUAD	Lung adenocarcinoma
UCS	Uterine Carcinosarcoma
MESO	Mesothelioma
CHOL	Cholangiocarcinoma
ESCA	Esophageal carcinoma
HNSC	Head and Neck squamous cell carcinoma
LUSC	Lung squamous cell carcinoma
UCEC	Uterine Corpus Endometrial Carcinoma
STAD	Stomach adenocarcinoma
STES	Stomach and Esophageal carcinoma
THYM	Thymoma
PCPG	Pheochromocytoma and Paraganglioma
LAML	Acute Myeloid Leukemia
GO	Gene Ontology
DEGs	Differentially Expressed Genes
BP	biological process
CC	cellular component
MF	molecular function
IC50	half maximal inhibitory concentration
FUSCC	Fudan University Shanghai Cancer Center
CNV	Copy Number Variation
CPTAC	Clinical Proteomic Tumor Analysis Consortium
DC	dendritic cell
RP	Regulatory Potential
KEGG	Kyoto Encyclopedia of Genes and Genomes
CCLE	Cancer Cell Line Encyclopedia
