# Supplementary figures and images for: The expression and prognostic value of transporter 1, ATP binding cassette subfamily B member in clear cell renal cell cancer with experimental validation
Source: Front Oncol. 2022 Nov 7;12:1013790. doi: 10.3389/fonc.2022.1013790 (PMC9676953; doi:10.3389/fonc.2022.1013790)

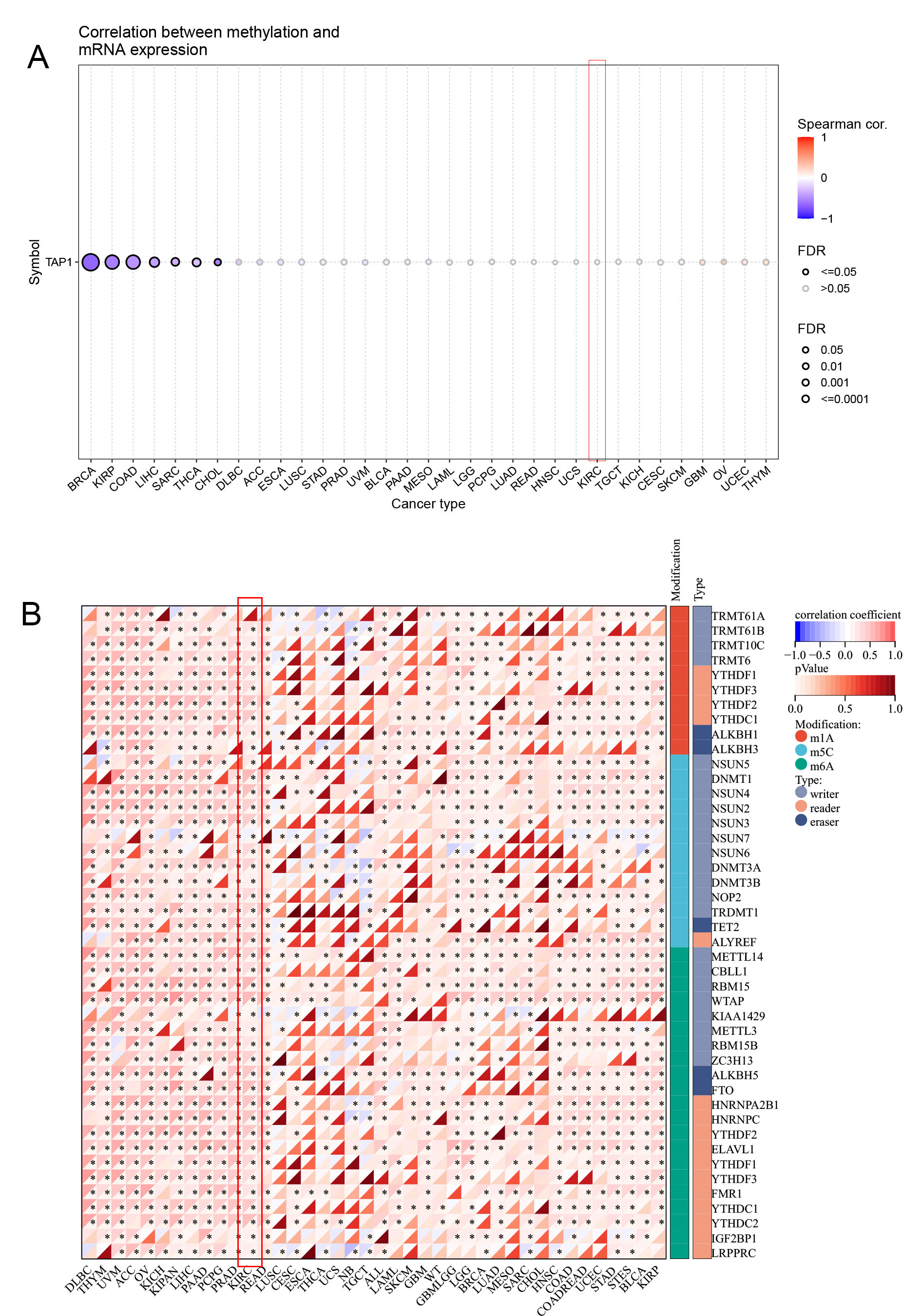

Supplement: Supplementary Figure 1 — DNA and RNA methylation modification in TAP1. (A) TAP1 expression is inversely related to DNA methylation in KIRC. (B) TAP1 expression is positively correlated with RNA methylation in KIRC. [file Image_1.jpeg]

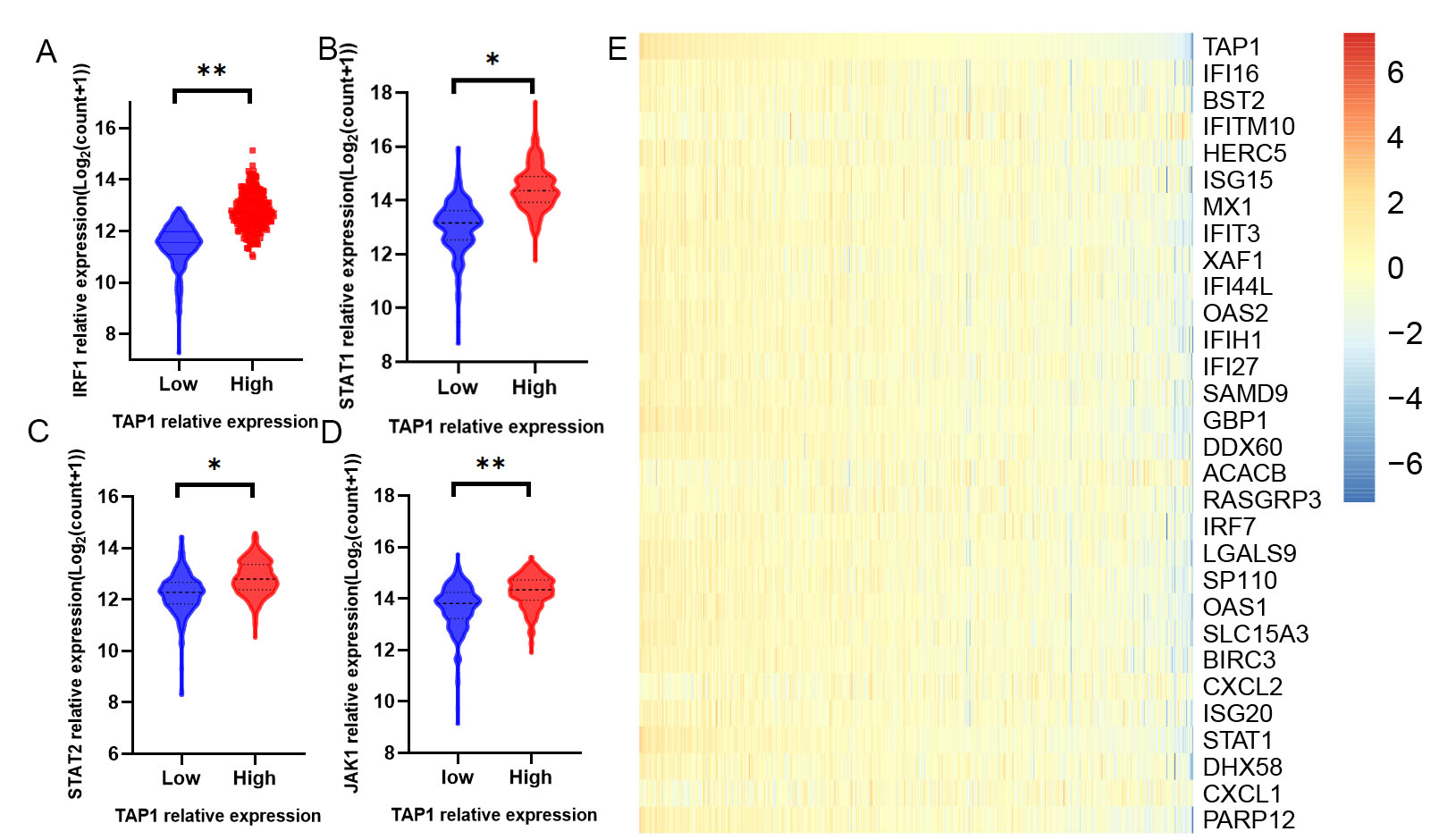

Supplement: Supplementary Figure 2 — TAP1 is positively correlated with interferon (IFN) signaling pathway. (A) IRF1 expression between low and high-TAP1 expression groups downloaded from the TCGA Database. (B) STAT1 expression between low and high-TAP1 expression groups downloaded from the TCGA Database. (C) STAT2 expression between low and high-TAP1 expression groups downloaded from the TCGA Database. (D) JAK1 expression between low and high-TAP1 expression groups downloaded from the TCGA Database. (E) Heatmap show the positive correlation of TAP1 expression with IFN response signature genes in the TCGA RNA-Seq Database of ccRCC. Statistical significance was determined by Pearson’s correlation test (*, P < 0.05; **, P < 0.01; ***, P < 0.001; ****, P < 0.0001). [file Image_2.jpeg]
